# Morphological and ultrastructural features of the laryngeal mound of Egyptian Cattle Egret (*Bubulcus ibis*, Linnaeus, 1758)

**DOI:** 10.1186/s40850-022-00147-4

**Published:** 2022-07-29

**Authors:** Fatma A. Madkour, Mohammed Abdelsabour-Khalaf

**Affiliations:** Department of Anatomy and Embryology, Faculty of Veterinary Medicine, South Valley University, Qena, 83523 Egypt

**Keywords:** Cattle egret, Laryngeal cartilages, Laryngeal salivary glands, Papillae

## Abstract

**Background:**

According to our acknowledgment this is the first full anatomical description of the studied laryngeal mound of the Egyptian Cattle Egret *(Bubulcus ibis*, Linnaeus, 1758). This study was obtained with the aid of scanning electron microscopy (SEM) and histological techniques. Heads of ten healthy adult male Egyptian cattle egrets were used in this study.

**Results:**

The laryngeal mound (*Mons laryngealis*) was a pear-shaped musculoskeletal elevation. It represented 20.55 and 67.87% of the total length of the oropharyngeal floor and the pharyngeal floor, respectively**.** By SEM view, the lateral aspect of the caudal third of the laryngeal mound had a serrated mucosal appearance, forming of 6–7 finger-like projections. Furthermore, the terminal part of the laryngeal mound (except the middle part) was bordered a transverse row of pyramidal-shaped papillae, which demarcated from the esophagus. Histologically, laryngeal salivary glands termed (cricoarytenoid salivary glands) of the laryngeal mound were simple tubular type and were arranged in one row within the lamina propria connective tissue close to the lamina epithelialis. Those glands were surrounded by abundant aggregation of lymphocytes, extended overlying the surface lining epithelium. The glottis within the laryngeal mound was supported by hyaline cartilages; dorsally by paired arytenoid cartilages, ventrolaterally by cricoid cartilage, and caudodorsally by procricoid cartilage. Two groups of intrinsic laryngeal skeletal muscles have connected the cartilages. The glandular epithelium of the laryngeal salivary glands and chondrocytes of the laryngeal cartilages showed strongly positive alcian blue reaction.

**Conclusions:**

The laryngeal mound shows certain features that are unique as an adaptation to lifestyles and bird’s habitat.

## Introduction

The cattle egret *(Bubulcus ibis,* Linnaeus, 1758), the multinational heron species (Family: Ardeidae, Order: Ciconiiforms) disperses in warm climates where its distribution is rapidly expanding. His Arabic name, Abu quirdan means “father of ticks”, a name emanated from the abundance of bird ticks grow in her spawning colonies [26].

In Egypt, usually inhabitant in the Nile Valley and Delta, nesting colonially in farms and wetlands on bushes with near water bodies [38]. It is a white bird decorated with buff feather during breeding season and has a sharply pointed yellow short beak. It sometimes accompany cattle or other large animals, capturing insects and small vertebrates, which are disturbed by these creatures, in addition to feeding on earthworms, lizards, crickets and flies in the earth [37].

The morphology and physiology of avian respiratory system differed distinctly from those of mammals [30, 32]. The laryngeal mound performs both respiratory and digestive functions so a variety of scholars have studied the anatomy of the laryngeal mound in various avian species such as Egyptian geese [29], turkey [4, 34], guinea fowl [13], Long-Legged Buzzard [15] and other birds [6, 27, 31]. We compared the nasal cavity of the aquatic and non-aquatic birds [20].

Very few morphological studies were performed on the laryngeal mound of cattle egret and none describing it microscopically or scanning electron microscopically (SEM). The present study aimed to provide a wide range of details morphological analysis of laryngeal mound of cattle egret from various perspectives, especially gross anatomy, light microscopic examination, in addition to scanning electron microscopy.

## Materials and methods

The present investigation was carried out under the supervision of the Animal Health and Ethics Committee of the Faculty of Veterinary Medicine, South Valley University and under Egyptian rule.

Ten healthy adult male Egyptian cattle egrets (375 g bwt) were captured from Assiut governorate (Egypt), were used in the current study. We customized five birds for the macro anatomical, two for the scanning electron microscopy, and three for the histological studies.

The birds were anaesthetized with 1:1 mixture of ketamine-xylazine (0.0044 cc/kg injected in the pectoral muscle), then euthanized and left to completely bleed. All methods are reported in accordance with ARRIVE guidelines.

The heads were collected and opened through one angle of the mouth to clarify the laryngeal mound. The laryngeal mound samples were dissected, and the gross characteristics, location, and shape were identified. The different measurements were taken including: the length of the oropharyngeal floor, pharyngeal floor, and laryngeal mound, as well as the length and width of the glottis by using Digital Vernier Caliper. Data were expressed as mean and stander deviation (SD).

The histological techniques are applied according to Ahmed et al. [2] and Madkour [21], the samples of the laryngeal mound were fixed in 4% buffered formaldehyde. The samples were dehydrated in a graded ascending ethanol series, then were transferred to xylene and embedded in paraffin wax. Paraffin sections were cut to a thickness of 3-5 μm and stained by with Hematoxylin and Eosin (H&E) and alcian blue (AB). All staining methods were approved after Bancroft and Gamble [7].

For scanning electron microscopy, the samples were submerged in 5% glutaraldehyde, then fixed in 1% Osmium tetroxide, dehydrated in alcohol, accompanied by amyl acetate and critical point dried using liquid CO2 and placed on sample stubs, sputter gold coated and investigated by using JEOL scanning electron microscopy (JSM-5400). According to previously published papers [17, 24, 23, 25], the scanning electron microscopic images have been digitally colored using the Photo Filter 7.2.1 program to be more visible to the readers.

## Results

### Gross anatomical and morphometrical features

**Fig. 1:**
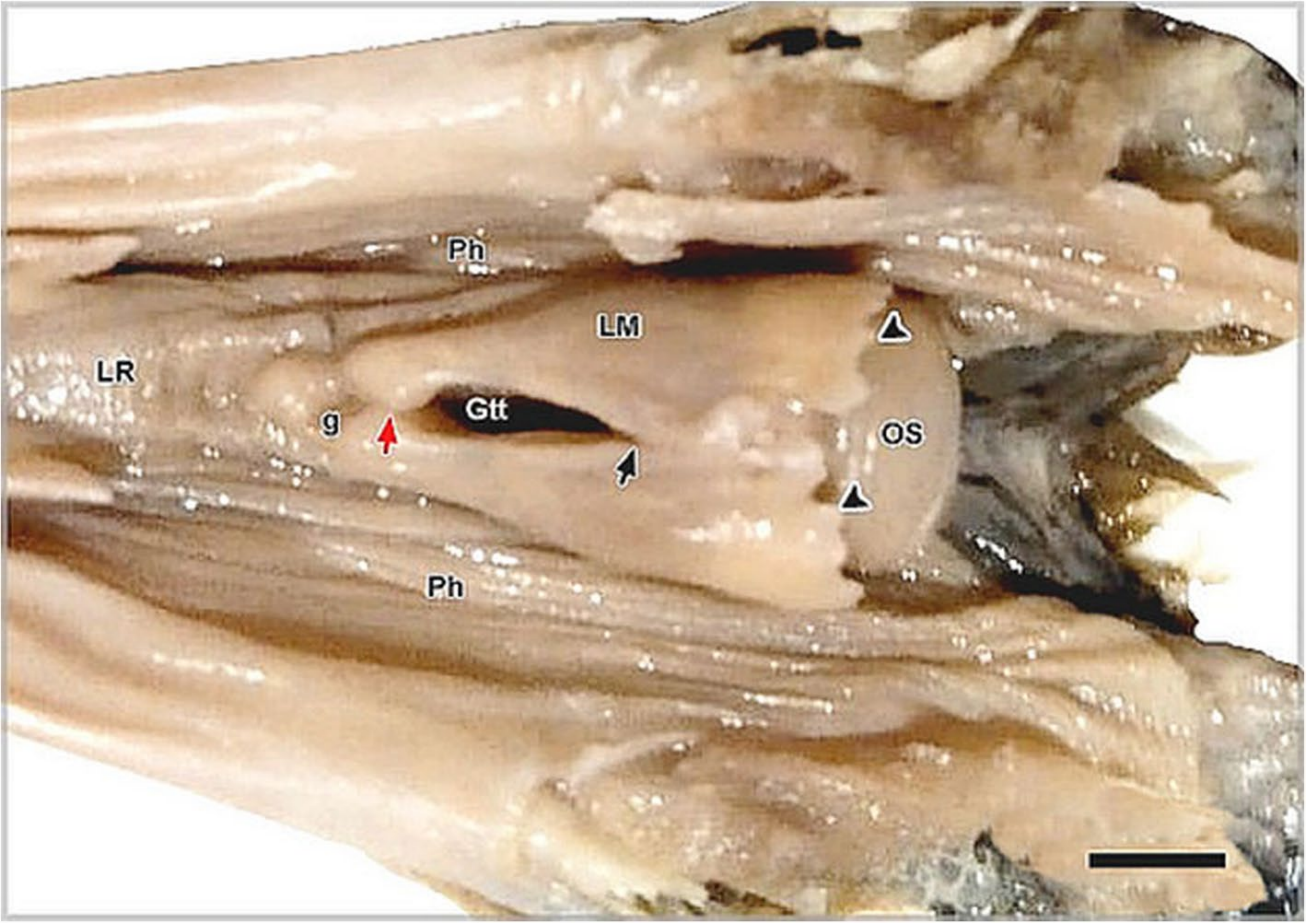
Photograph of the laryngeal mound (LM) of Cattle Egret within the pharyngeal floor (ph), located caudal to the lingual root (LR). Showing glottis (Gtt) accompanied by rostral laryngeal sulcus (red short arrow) with deep groove (g), and shallow laryngeal sulcus (short arrow), a transverse row of papillae (arrowheads) demarcated from the esophagus (OS). Scale bar = 1 cm

The laryngeal mound (*Mons laryngealis*) was a pear-shaped musculoskeletal elevation projected within the caudal part of the pharyngeal floor, caudal to the lingual root (Fig. 1). Its length measured 15.53 ± 0.79 mm, represented 20.55 and 67.87% of the total length of the oropharyngeal floor and the pharyngeal floor, respectively. The width of the laryngeal mound was 2.24 ± 0.14 mm at its cranial end and 7.69 ± 0.71 mm at its caudal end.

The laryngeal mound contained an elongated opening called glottis. The glottis measured 5.84 ± 0.43 mm long, continued rostrally by rostral laryngeal sulcus (1.26 ± 0.24 mm long), and caudally by shallow laryngeal sulcus (3.43 ± 0.16 mm long). The rostral laryngeal sulcus was marked cranially by slightly deep groove. The caudal part of the laryngeal mound was demarcated from the esophagus by a transverse row of papillae (Fig. 1). The morphometrical measurements of the laryngeal mound of Egyptian Cattle Egret were recorded in Table 1.

**Table 1:** Morphometrical measurements of the laryngeal mound of Egyptian Cattle Egret

Dimensions	Mean	SD
- Length of oropharyngeal floor	75.57	5.20
- Length of pharyngeal floor	22.88	1.70
Laryngeal mound:		
-Length of:		
- Laryngeal mound	15.53	0.79
- Laryngeal inlet (glottis)	5.84	0.43
- Rostral laryngeal sulcus	1.26	0.24
- Caudal laryngeal sulcus	3.43	0.16
-Width of:		
- Laryngeal mound at its cranial end	2.24	0.14
- Laryngeal mound at its caudal end	7.69	0.71
- Laryngeal inlet (glottis)	1.18	0.25

*Note:* All measurements (mm) were expressed in (mean ± SD)

### Microscopical observations

**Fig. 2:**
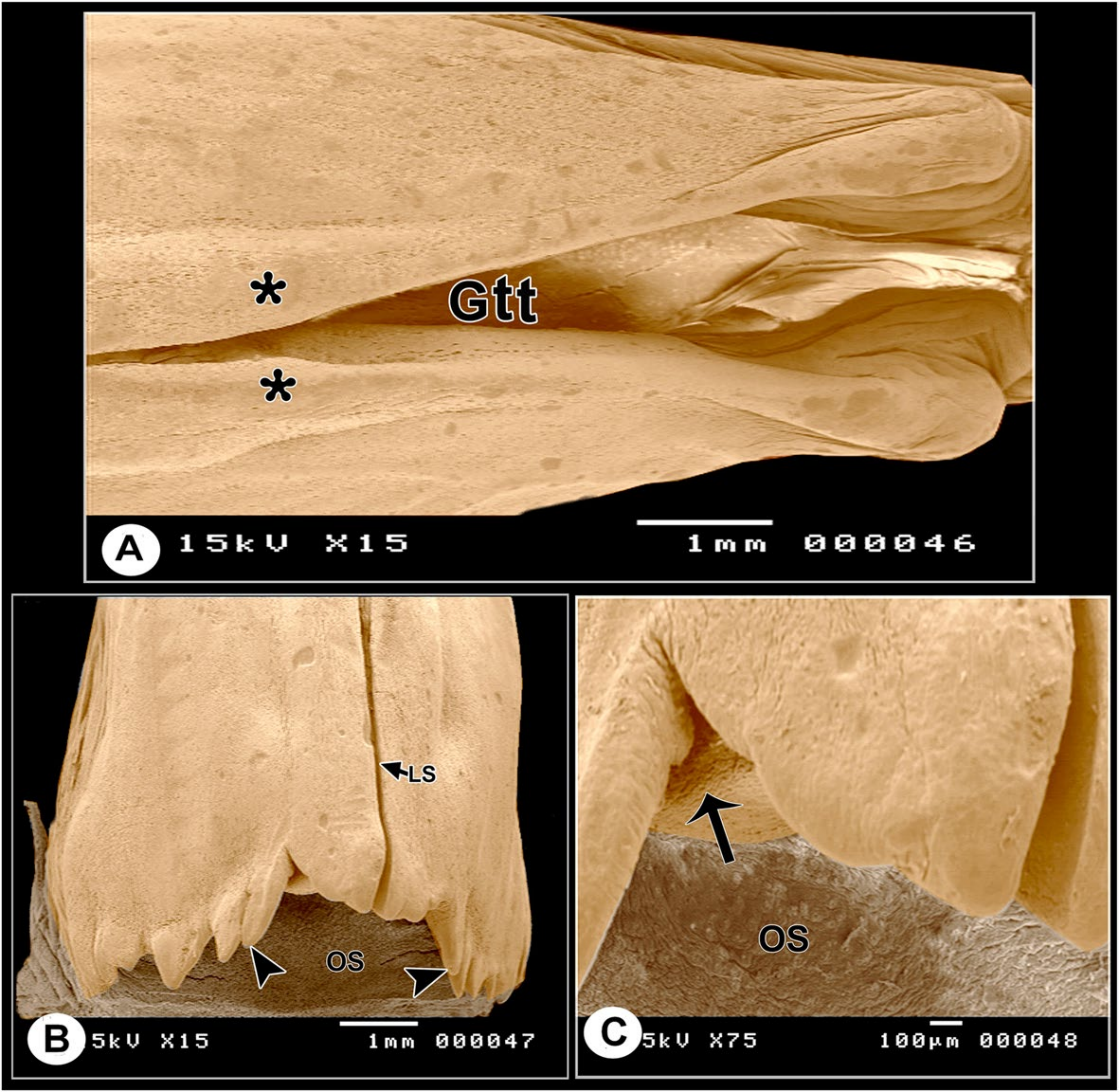
Colored scanning electron micrographs of the laryngeal mound of Cattle Egret, showing glottis of the laryngeal mound (Gtt) with its rims had mucosal lip (asterisks), elongated slit-like caudal laryngeal sulcus (LS), the terminal part of the laryngeal mound (except middle part) was characterized by a transverse row of pyramidal-shaped papillae (arrowheads), which demarcated from the esophagus (OS), mucosal sheet (arrow)

By SEM view, the edges of the laryngeal mound were thickened, smooth, marked by mucosal lips. These lips were mostly observed at the rostral end and middle part of the glottis, as well as they were slightly elevated above the surface of the laryngeal mound (Fig. 2A). The glottis continued caudally by an elongated slit-like sulcus (caudal laryngeal sulcus). Moreover, the terminal part of the laryngeal mound (except the middle part) was bordered a transverse row of pyramidal-shaped papillae, which demarcated it from the esophagus. These papillae were increased in size lateral wards (Fig. 2B). Mucosal sheet was paramedian to the caudal end of the sulcus (Fig. 2C).

**Fig. 3:**
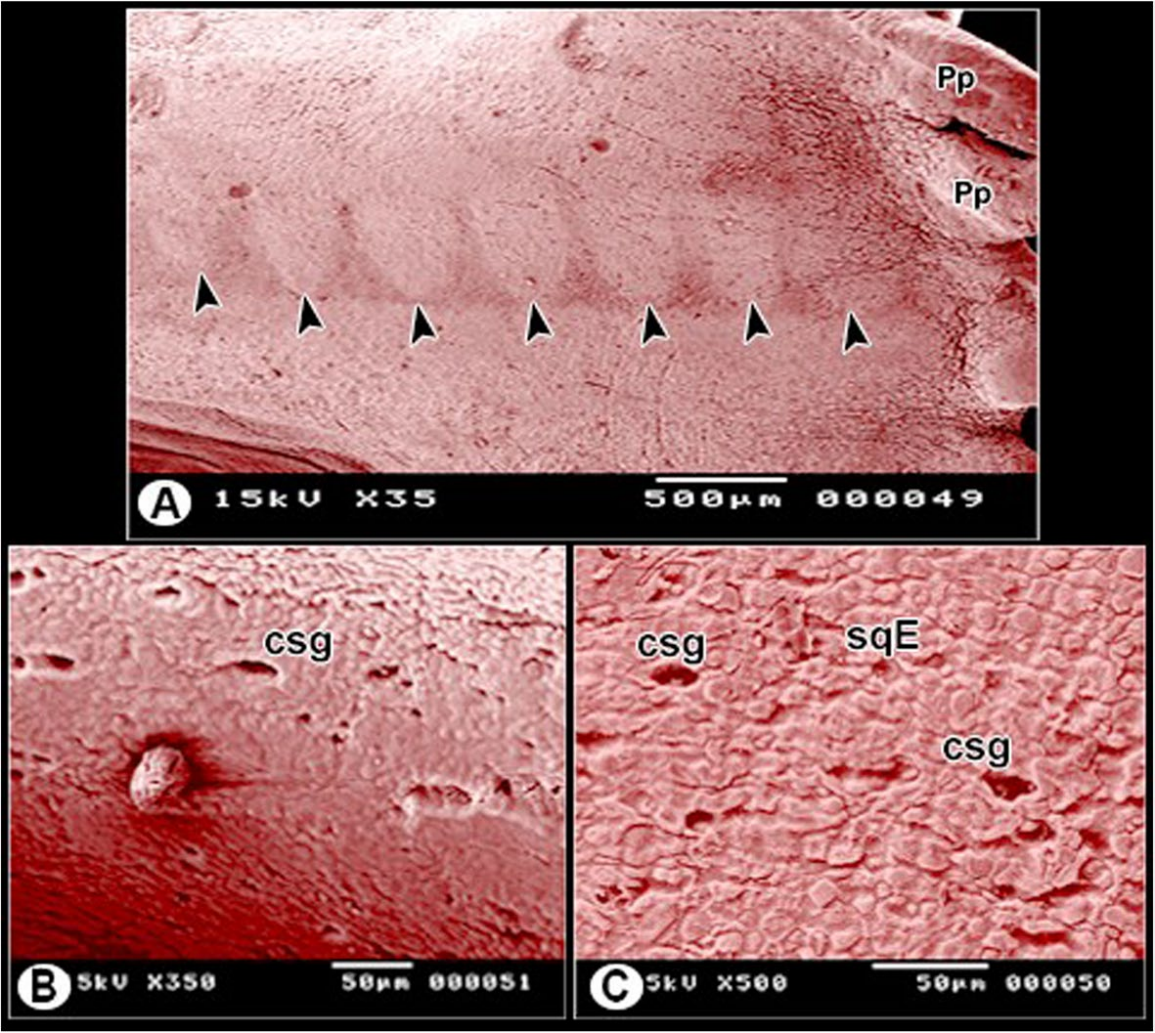
Colored scanning electron micrographs of the caudolateral part of the laryngeal mound of Cattle Egret, showing serrated mucosal appearance forming of 6–7 finger-like projections (arrowheads), various sizes of the openings of the cricoarytenoid salivary glands (csg), lining stratified squamous epithelium (sqE) of the laryngeal mound, pyramidal-shaped papillae (Pp)

The lateral aspect of the caudal third of the laryngeal mound had a serrated mucosal appearance, forming of 6–7 finger-like projections (Fig. 3A). By high magnification, various sizes of the openings of the cricoarytenoid salivary glands were observed within the non-cornified stratified squamous lining epithelium (Fig. 3B). This epithelium was lined the laryngeal mound till the edges of the glottis which represented the continuation of the lining epithelium of the pharynx (Fig. 3C).

**Fig. 4:**
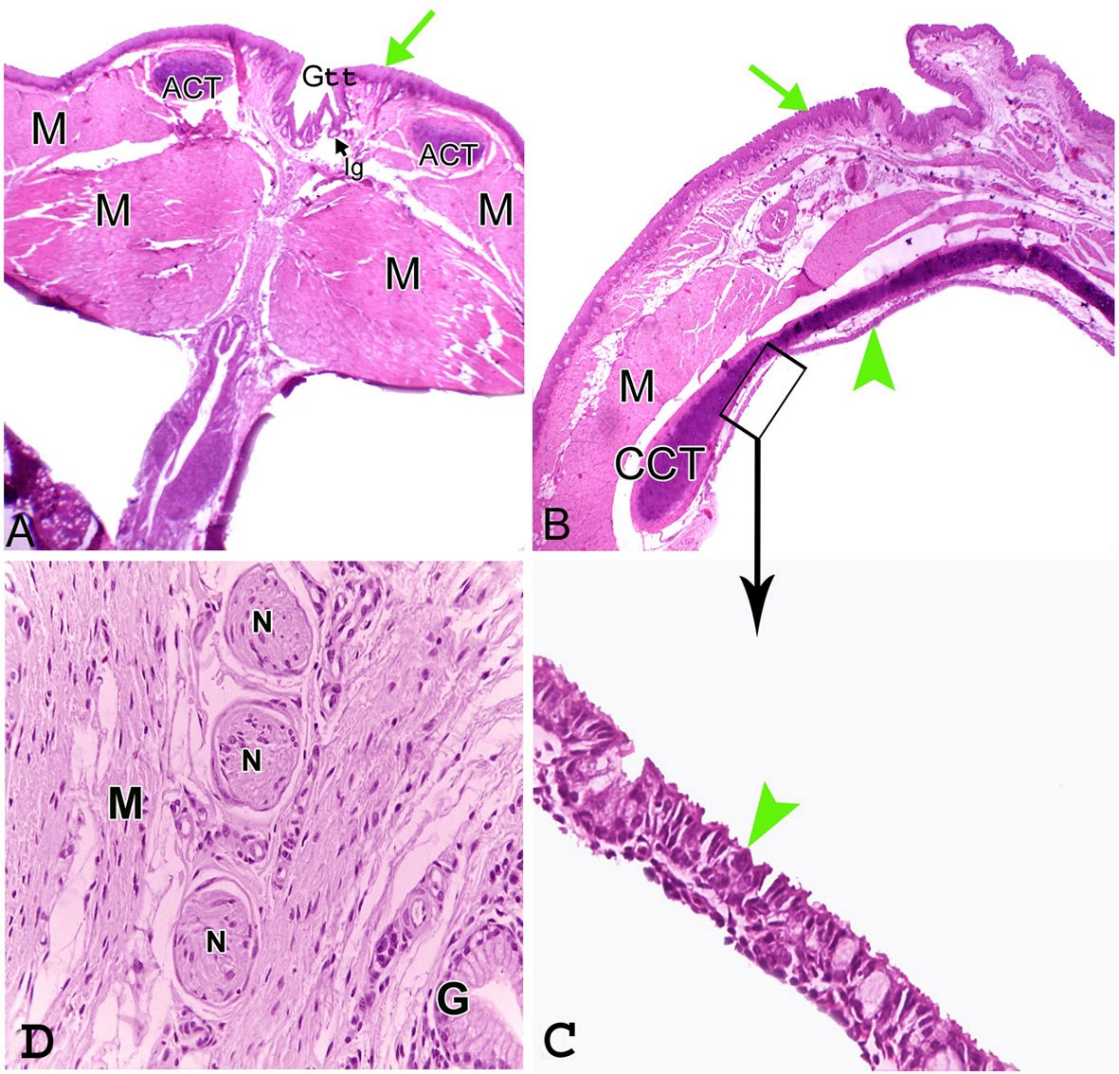
Photomicrographs of the cross-section of the rostral part of the laryngeal mound of Cattle Egret, showing stratified squamous epithelium (green arrow) interrupted by intraepithelial mucous glands (Ig) mostly noticed at the glottis (Gtt), and transformed into ciliated pseudostratified columnar epithelium (respiratory epithelium) (green arrowhead), paired arytenoid cartilages (rostral process) (ACT) supported the laryngeal inlet, and cricoid cartilage (CCT), these cartilages were supported by two layers of intrinsic laryngeal muscles (M), peripheral nerve endings (N) with the skeletal muscle fibers beneath salivary glands (G). H&E stain with X40(View **A**), X100(View **B**), X400, and (Views **C**, **D**)

**Fig. 5:**
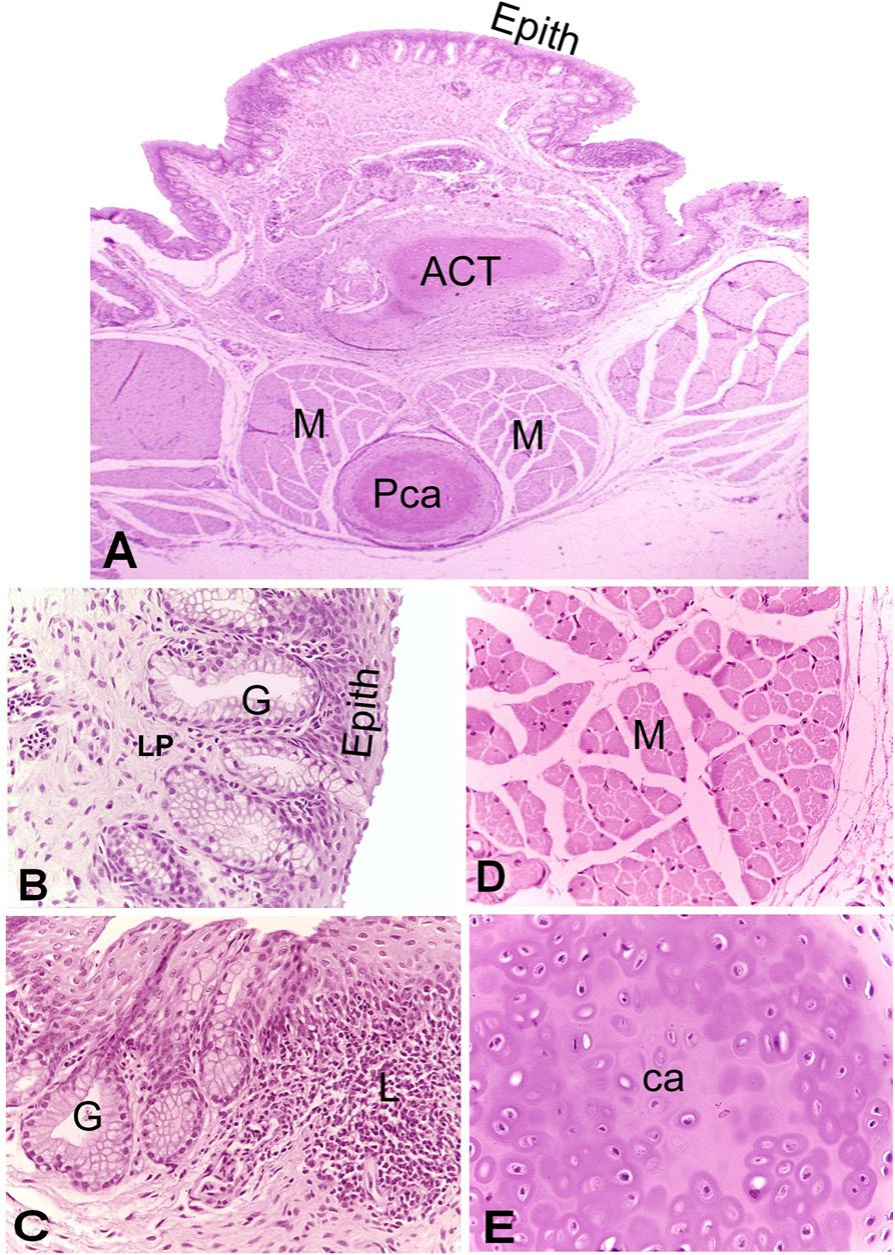
Photomicrographs of the cross-section of the caudal part of the laryngeal mound of Cattle Egret, showing cricoarytenoid salivary glands (G) within the lamina propria connective tissue (LP) close to stratified squamous epithelium (Epith), surrounded by abundant aggregation of lymphocytes (L), the rostral process (ACT) of the arytenoid cartilage, procricoid cartilage (Pca), two spherical masses of intrinsic laryngeal skeletal muscles (M), chondrocytes located inside the lacunae and surrounded by matrix (ca). H&E stain with X100(View **A**), and X400(Views **B**-**F**)

By light microscopical view, the non-cornified stratified squamous lining epithelium of the laryngeal mound was interrupted by intraepithelial mucous glands, which were more noticed at the median part toward the glottis (Fig. 4A). The non-cornified stratified squamous epithelium of the dorsal surface of the laryngeal mound was the continuation of the lining epithelium of the pharynx till the edges of the glottis, then transformed into ciliated pseudostratified columnar epithelium (respiratory epithelium) (Fig. 4B, C). Aggregations of peripheral nerve endings were associated with skeletal muscles fibers beneath mucous laryngeal salivary glands (Fig. 4D). The laryngeal salivary glands (cricoarytenoid salivary glands) were arranged in one row within the lamina prorpia connective tissue close to the lamina epithelialis (Fig. 5A).

Most of the laryngeal salivary glands (cricoarytenoid salivary glands) were simple tubular type and penetrated the mucosa to open on the surface of the laryngeal mound into the pharyngeal cavity. Each gland was lined by low columnar mucous secreting cells with rounded basally located nuclei. These cells had foamy, vacuolated, faintly stained basophilic cytoplasm (Fig. 5B). The aforementioned glands were surrounded by abundant aggregation of lymphocytes as well as the lymphoid tissue was extended overlying the surface lining epithelium (Fig. 5C).

**Fig. 6:**
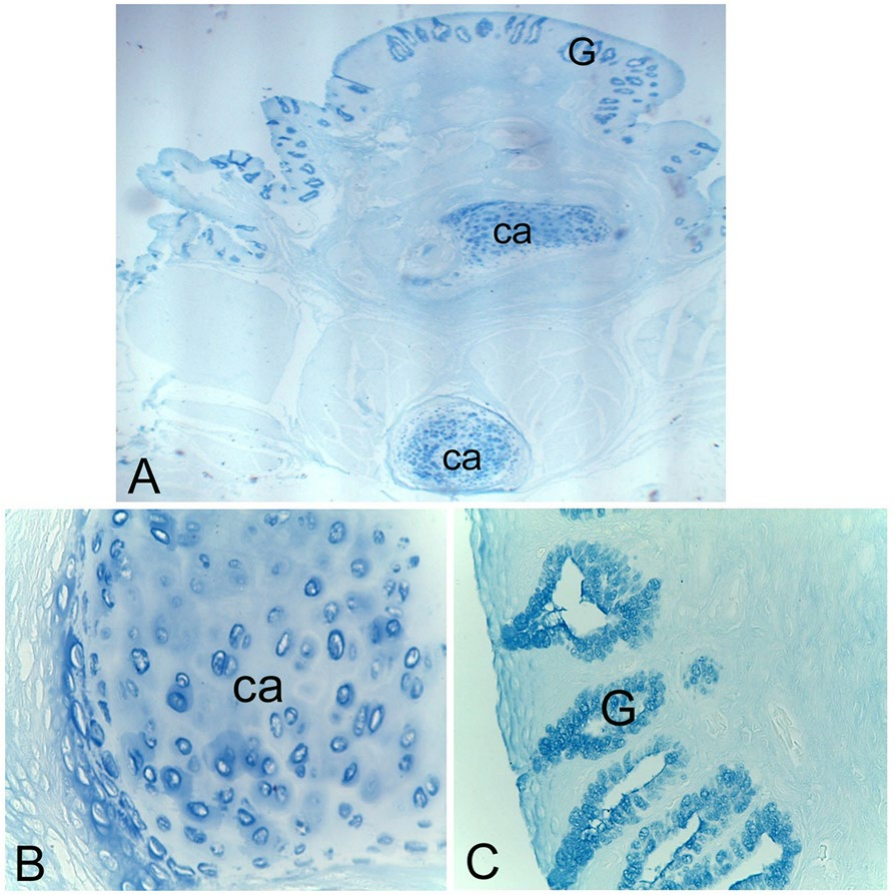
Photomicrographs of the cross-section of the caudal part of the laryngeal mound of Cattle Egret. Chondrocytes (ca) of the laryngeal cartilages and the lining epithelium of the laryngeal salivary glands showing strongly positive alcian blue reaction. Alcian blue stain X40 (View **A**), and X400 (**B**, **C**)

The glottis was supported by hyaline cartilages; dorsally by paired arytenoid cartilages, ventrolaterally by cricoid cartilage, and caudodorsally by procricoid cartilage (Figs. 4A & 5A). Two groups of intrinsic laryngeal skeletal muscles connected the cartilages. One group of those muscles appeared as two spherical masses on each side of the procricoid cartilage (Figs. 4A& 5A, D). The chondrocytes of the laryngeal cartilages were located inside the lacunae and surrounded by matrix (Fig. 5E). The glandular epithelium of the laryngeal salivary glands and chondrocytes of the laryngeal cartilages showed strongly positive alcian blue reaction (Fig. 6A-C).

## Discussion

The anatomical description of Egyptian Cattle Egret has received little attention from anatomists in comparison with other avian species. It is importance to focus on the anatomical descriptions of its laryngeal mound, which is a crucial section of its respiratory system to be beneficial for manipulation of head during treatment of diseases especially respiratory one.

Several works of studies supported our findings that the laryngeal mound is a raised structure located caudal to the lingual root [1, 14, 18, 19, 29]. The laryngeal mound of cattle egret was pear-shaped. It is an elongated heart-shaped in turkey [4], pyramidal-shaped in broad breasted white turkey [19], a small mound in Long-Legged Buzzard [15], triangular shape in laughing dove and like heart on playing card in Japanese quail [18].

The morphometrical study recorded that the laryngeal mound was 15.53 mm long and 2.24, 7.69 mm wide at its cranial and caudal parts, respectively. However, Al-Ahmady [5] clarified that the length of the laryngeal mound of the same studied bird is 7 mm and its width is 1.5 mm at the middle part. In laughing dove and Japanese quail, the length is 5.99, 6.40 mm, respectively [18]. The latter author [19] showed that the laryngeal mound of broad breasted white turkey is 34.81 mm long and 17.81 mm wide at the cranial ends and 22.52 mm at the caudal ends.

In the present study as well as Madkour [18] in Japanese quail, the edges of the laryngeal mound were marked by mucosal lips which were slightly elevated above the surface. Moreover, the edges were smooth free from papillae as mentioned by the latter author in laughing dove. In contrast, each rim of the laryngeal mound is guards by sagittal row of caudally directed papillae as described by Mohamed et al. [29] in Egyptian geese. Many conical papillae are bounding the glottis in raven and magpie [11]. By SEM view, we showed a peculiar feature on the lateral aspect of the caudal third of the laryngeal mound, which had 6–7 finger-like projections giving a serrated mucosal appearance.

In the same line several researchers identified that the caudal part of the laryngeal mound was bordered many papillae arranged in rows. A transverse row of pyramidal-shaped papillae is observed in the examined bird, in red jungle fowl [16], and in hoopoe [1]. Moreover, in this respect, the caudal part of the laryngeal mound is characterized by a conical papillary row in southern lapwing [12], V-shaped row pyramidal-like papillae regarding dove, and three transverse rows of conical shaped papillae regarding Japanese quail [18]. Kabak et al. [15] clarified two rows of the lateral papillae on either sides of laryngeal groove in Long-Legged Buzzard. Whilst, numerous irregular distributed papillae occupied the caudal part of the laryngeal mound in Egyptian geese [29]. Furthermore, Al-Ahmady [5] showed that the laryngeal mound of egret cattle is bounded caudally by a single row of backwardly and linearly arranged large sized pharyngeal papillae, four on each side. These papillae play role in passage of the bolus toward the esophagus.

Our histological observations showed that the lining epithelium of the laryngeal mound was non-cornified stratified squamous epithelium till the laryngeal inlet. This result agrees with the reports of many published data in other different birds such as Egyptian geese [29], laughing dove and Japanese quail [22], duck [28], and turkey [3, 34]. The latter author only showed the stratified squamous epithelium is keratinized, which is different according to the feeding habits of the birds. The epithelium is withstanding the friction of the food so be different according expose to the greatest amount of friction during feeding.

The current study revealed that the cricoarytenoid salivary glands were numerous distributed in one row along lamina propria closely to the lamina epithelialis. In this point, Madkour [22] reported that the cricoarytenoid salivary glands are arranged in three groups in Japanese quail and two groups in laughing dove, while Mohamed et al. [29] in Egyptian geese and Mohamed [28] in duck showed that the laryngeal mound has four groups of these glands. On other hand, in emu, the glands concentrated on the dorsolateral aspect of the arytenoid cartilages, and the rest of the laryngeal mound are free of glands [10].

The cricoarytenoid salivary glands were tubular type that boosted the suggestions of several authors who found that the most common type of glands in birds is tubular type [8, 9, 33, 36]. In the studied Cattle Egret, mucous secreting cells of the cricoarytenoid salivary glands showed a strongly positive Alcian blue reaction. This result indicated that the mucous secreting cells secrete acidic mucopolysaccharides. However, Saleh [34] mentioned that these cells show strongly positive PAS reactions. Moreover, Mohamed et al. [29] reported that the cricoarytenoid salivary glands secrete both acidic and neutral mucopolysaccharides. Mucins in saliva protect mucosal surfaces from toxin and mechanical damage, assist in maintaining cellular water balance, provide lubrication, and have an antimicrobial action [10, 35]. Contrary to the previously mentioned data, we observed in the studied bird that the cricoarytenoid glands are surrounded by abundant lymphoid tissue.

The present study revealed that the laryngeal cartilages of the laryngeal mound were a hyaline type, the chondrocytes were located inside the lacunae and surrounded by matrix. In this connection, the body of the arytenoid and procricoid cartilages are ossified and become trabeculae in Japanese quail [22]. Moreover, the cricoid cartilage is fully ossified in turkey [3]. The ossification of these cartilages in some birds is considered as strong prop of the larynx.

In conclusion, this study provides comprehensive information on the macro/micro-structures of the laryngeal mound of Egyptian Cattle Egret. Further studies are required to have a complete local atlas for the anatomy of this bird.

## Data Availability

The datasets generated and/or analyzed during the current study are not publicly available due to the rules of our institute but are available from the corresponding author on reasonable request.
